# Effects and Implications of COVID-19 for the Human Senses, Consumer Preferences, Appetite and Eating Behaviour: Volume I

**DOI:** 10.3390/foods11121738

**Published:** 2022-06-14

**Authors:** Derek Victor Byrne

**Affiliations:** 1 Food Quality Perception and Society Science Team, iSense Lab, Department of Food Science, Faculty of Technical Sciences, Aarhus University, DK-8200 Aarhus, Denmark; derekv.byrne@food.au.dk; 2 Food & Health Research, Sino-Danish Center (SDC), Niels Jensens Vej 2, DK-8000 Aarhus C, Denmark

## 1. Introduction

Severe Acute Respiratory Syndrome Coronavirus-2 (SARS-CoV-2) evolved into a global pandemic in 2020 [1]. The assessment of coronavirus disease 19 (COVID-19) patients has presented a health condition including, in many cases, a mild to severe loss of smell and tasting abilities among patients, see, e.g., [2,3].

Initial work has shown short- and likely longer-term negative effects on the human senses, with some indications for effects on consumer preferences; however, as of yet, very little is known about the impacts on eating behaviours and consequent longer-term effects on appetite (see [4]).

Food enjoyment is a key aspect of people’s appetite, and any loss in expected pleasure greatly affects our motivation to eat, potentially leading to persons affected by COVID-19 to experience core changes in relation to their food intake practices, which may potentially have long-term implications for health and recovery [5].

The aim of this Special Issue was, for the first time, to bring together researchers with key insights on how COVID-19 has impacted appetite and eating behaviours, from the fundamental to the applicable, as assessed by human sensory perception and behaviour [6,7,8,9,10,11,12,13,14,15,16,17].

Through this call for publications, we wished to document and bring together ongoing key research in order to ensure that this research has a lasting impact regarding our future understanding of the measures developed to help and treat people affected during the ongoing pandemic.

Moreover, we requested the inclusion of a range of research from fundamental effects on the senses, to changes in consumer preferences all the way to how and why COVID-19 has changed consumer behaviours in relation to food and eating in the longer term [6,7,8,9,10,11,12,13,14,15,16,17].

The articles included have been categorized based on their core aims and findings, and they cover research in relation to COVID-19 and the senses in four key areas, with respect to appetite and eating behaviour [6,7,8], food choice and preference [9,10,11], the perception of food risk and safety, [12,13,14] and finally to the effects on purchasing behaviour during and after the initial waves of the COVID-19 pandemic [15,16,17]. This collection of articles is, in essence, a time capsule of the wide focus and importance of sensory and consumer science in the COVID-19 space thus far, and its highlighting of effects on societies senses, eating practices and behaviours.

Ultimately, the goal was to publish the Special Issue collection as an open-source book to act as a tool for understanding the long-term effects of COVID-19 on human health related to food and eating issues. This endeavour is now complete with this group of publications, now designated as Volume I on the ‘Effects and Implications of COVID-19 for the Human Senses, Consumer Preferences, Appetite and Eating Behaviour’. Due to a continuously growing body of work on COVID-19 being submitted to the Sensory and Consumer area of *Foods*, and to ensure we place a focus on the important work on COVID-19 in the sensory sphere, we have determined that a Volume II of this book will be curated and published in due course.

## 2. A Synopsis of Special Issues Research

### 2.1. Appetite and Eating Behaviour Change

Thus, in relation to appetite and eating behaviour change, authors Høier et al. (2021) investigated subjective strategies for maintaining appetite in recovering COVID-19 patients [15]. The study involved in-depth interviews, focusing on patients suffering from the long-term effects of COVID-19. The results were analysed using a thematic analysis for qualitative data. The results on strategies for maintaining appetite included a focus on well-functioning senses, a focus on familiar foods, a focus on the eating environment, and finally a focus on post-ingestive well-being. Høier et al. (2021) found that factors prior to, during and after food intake, as well as the context, could influence both the desire to eat and the pleasure related to food intake [15]. Moreover, the authors indicated that, as ageusia and anosmia make the characterization of food difficult, being able to recognize and memorize its flavour was important to engage in consumption; under normal circumstances, the hedonic value of food relies predominantly on the flavour of the food. When suffering from chemosensory dysfunction, shifting focus towards the texture of food, including trigeminal stimulation during consumption, was beneficial for maintaining appetite and food-related pleasure. Furthermore, a focus on the holistic satisfying feelings of choosing healthy food, as well as a focus on other people’s enjoyment during meals, were reported to boost well-being around food intake. Høier et al. (2021) concluded that research elaborated our understanding of the complex consequences of COVID-19 and can be applied in health-promoting initiatives targeting patients recovering from COVID-19 [15].

Furthermore, regarding changes in eating behaviour, Chaaban et al. (2021) investigated the acute and long-term effects of COVID-19 disease on the desire for food, hunger, and satiety sensations; smell, taste, and flavour perception; meals and intake of food types; and the frequency of commonly applied strategies to tackle potential changes in appetite and sensory perception [14]. In this study, an online survey was conducted among Danish adults who had experienced changes in appetite, sensory perception, and/or food-related pleasure due to COVID-19 [14]. The overall results indicated appetite-altering effects at all times during the day when suffering from COVID-19 and were often associated with impaired sensory function. The authors also showed severe sensory perception alterations, namely, for the perception of taste and for the perception of smell. Eating behavioural changes included alteration in quantitative and qualitative aspects of intake. The effects were, in general, more pronounced during the acute phase of disease than during the post-acute phase. Chaaban et al. (2021) concluded that the findings illustrate the complexity by which COVID-19 affects human appetite, sensory perception, and eating behaviour, but also point to strategies to cope with these changes [14].

Finally, in relation to appetite, Parker et al. (2022) looked at parosmia (a distortion in sense of smell) and its effects on food perception as a result of COVID-19. The aim of this study was to identify the key food triggers of parosmic distortions and investigate the relationship between distortion and disgust in order to establish the impact of parosmia on diet and quality of life. The authors indicate that olfactory dysfunction is amongst many symptoms of long COVID [6]. The authors contend that whilst most people that experience smell loss post COVID-19 recover their sense of smell and taste within a few weeks, around 10% of cases experience long-term problems, and their smell recovery journey often begins a few months later [6]. In the authors’ cross-sectional study, respondents experiencing smell distortions completed a questionnaire covering aspects of smell loss, parosmia and the associated change in valence of everyday items. It was determined that there was a significant correlation between strength and disgust and when the selected items were reported as distorted—they were described as either unpleasant or gag-inducing 84% of the time. The authors concluded that this shift in valence associated with loss of expected pleasure and the presence of strange tastes and burning sensations could certainly lead to changes in eating behaviours and serious longer-term consequences for mental health and quality of life [6].

### 2.2. Food Choice and Preference Effects

In relation to food choice and preference changes as a result of COVID-19, Scacchi et al. (2021) investigated how the Italian lockdown affected self-perceived food purchases (FP), occurrence of impulse buying (IB), and household food waste production (HFWP) as well as their respective determinants. A cross-sectional survey was distributed in May 2020, collecting an opportunistic sample of the Italian population. Most of the sample increased overall FP (53.4%) and food consumption (43.4%), and reduced HFWP (53.7%) and halved the prevalence of IB (20.9%) compared to the period before the lockdown (42.5%) [16]. Baking ingredients, fresh vegetables, fresh fruit and chocolate had the largest sales increase by individuals, while bakery products, fresh fish and salted snacks purchases highly decreased. Increased FP were associated with the occurrence of IB and inversely associated with not having worked during lockdown. Multivariable logistic regressions revealed that the occurrence of IB was associated with low perceived dietary quality according to the Emotional Overeating Questionnaire, and inversely associated with decreased HFWP. Reduced HFWP was associated with higher perceived dietary quality and negatively associated with a low score WHO-5 Well-Being Index [16]. Scacchi et al. (2021) concluded that the Italian lockdown highly affected FC behaviours, leading to positive and more sustainable habits towards food purchases and consumption. Public health interventions are needed to keep these new positive effects and avoid negative consequences in case of future lockdowns

In a study investigating trends in coffee and tea consumption during the COVID-19 pandemic, Castellana et al. (2021) mentioned that over the initial two years of the pandemic, many countries have enforced confinement to limit both the spread of COVID-19 and the demand for medical care. Confinement has of course resulted in a disruption of work routines, boredom, depression, and changes in eating habits, as well as changes in the consumption of coffee and tea. The authors indicate that beverage choices also contribute to daily calorie intake and hydration, particularly so-called ‘nervine’ beverages such as coffee and tea, in view of their purported potential to promote psychological well-being [17]. Castellana et al. (2021) investigated a large body of published studies in a database study, examining articles tracking consumption of tea and coffee. The authors found studies that indicated coffee consumption increased to some degree and tea consumption clearly increased. The authors indicate that the lack of a strong trend in coffee consumption as the result of the COVID-19 pandemic calls for additional investigation. Moreover, the authors state that potential health implications should not be overlooked, especially since caffeine consumption may directly or indirectly promote bronchodilation, interfere in the process of immunomodulation, and hinder viral intracellular transcription while undergoing COVID-19 infection [17]. In relation to tea, reflecting perhaps a discomfited mood and the socially confining setting, the authors found a marked increase in tea consumption. Tea is usually linked to routine and ritualized household consumption. Tea is historically instrumental in bringing the family closer together and provides a platform for sharing. In contrast, coffee consumption needed to be considered in a social, aesthetic and emotional context. Therefore, Castellana et al. (2021) conclude that setting aside the social context, the increased consumption of tea should be understood in emotional and family-related settings. From this perspective, this beverage has long been associated with mood and performance enhancements, such as a greater relaxation and concentration [17].

Finally, in relation to studies focused on food choice and preference changes as a result of the COVID-19 pandemic, Górska et al. (2021) indicated that psychological factors and restrictions imposed due to the pandemic may influence eating behaviours and physical activity. With the above thesis in mind, questionnaire-based surveys were conducted amongst residents of five European countries: Poland, Italy, Spain, Portugal and Great Britain (England and Scotland). A structured questionnaire was used to conduct anonymous internet surveys in 2020. It contained questions pertaining to sociodemographic data, eating behaviours, the impact of the pandemic on the diet and physical activity [11]. The questionnaire was made available to Internet users across the five countries. Górska et al. (2021) found that age was the parameter that impacted changing eating behaviours to the largest extent during the pandemic. It was also found that during the pandemic, regular consumption of meals was most dependent on various factors. The negative impact of the pandemic within this scope was most profound amongst women, city residents regardless of gender and people over 35 years of age. A change in the frequency of consumption of selected product groups during the pandemic was also observed. A reduced consumption of meat and fish was identified [11], in particular, among people under 35 living in Portugal, where almost half declared lower consumption of meat, and more than half reported lower consumption of fish. In an analysis of the results, the authors also showed an increase in the consumption of products with lower nutritional values, particularly amongst people under 35 years of age and also amongst residents of Great Britain (regardless of age). Moreover, the authors indicated that results showed that the pandemic may have had an impact on weight reduction [11]. Górska et al. (2021) survey results showed that the impact of the pandemic on eating behaviours was particularly profound when it came to meal consumption regularity. Changes to the consumption of products with lower nutritional values, which may decrease immunity, were also found during the pandemic. In conclusion, Górska et al. (2021) showed that the problem associated with consuming products with lower nutritional values was particularly evident amongst people under 35. Considering the global character of SARS-CoV-2 transmission, further research is necessary to determine its impact on the diet, nutritional status and physical activity [11].

### 2.3. Food Risk and Safety Perception

In a study focusing on food risk and safety perception as a result of COVID-19, Vandenhaute et al. (2022) looked specifically at safety measures in the food service sector and consumers’ attitudes and transparency perceptions at three different stages of the pandemic. Vandenhaute et al. (2022) indicate the study aims to examine consumers’ attitudes towards, and transparency perceptions of, COVID-19-related safety measures and to identify determinants of consumers’ intentions and behaviours regarding visiting restaurants and bars once reopened [7]. By surveying food service businesses in Belgium both during and in between waves of infections, the authors’ study allowed for a comparison between both target groups, i.e., 1697 consumers and 780 businesses. Vandenhaute et al. (2022) describe that the findings demonstrate that consumers evaluated safety measures as important when revisiting restaurants and bars, against business owners’ expectations [7]. Both consumers’ revisit intentions and behaviours are influenced by the perceived importance of hygiene measures (negatively) and past visit frequency (positively). These authors concluded that the study highlights the importance of good compliance with safety measures as a strategy to attract customers during the reopening period. Further, the findings emphasize the importance of transparent communication by food service businesses and the government [7].

Moreover, Cantalapiedra et al. (2022) in their paper, “Facing Food Risk Perception: Influences of Confinement by SARS-CoV-2 Pandemic in Young Population”, indicate a new food safety level of trust in food risk perception has been noticed, as a consequence of the SARS-CoV-2 pandemic. The pandemic encouraged a review of nutritional recommendations for the population, mainly for the young population. Here, the results of a survey designed for a young population, from the University of Valencia, Spain, in the health branch, and in charge of carrying out the shopping task for their household, were reported. The study reports three different scenarios and years, as defined by the SARS-CoV-2 pandemic: before the pandemic (the period of January–December 2019), during the pandemic lockdown (the period of March 2020–August 2020), and after the pandemic lockdown (the period of September 2020–June 2021) [8]. Cantalapiedra et al.’s (2022) survey was designed with questions, profiling responses using the best–worst elicitation (BWE) format. Results reported that trust and evaluation of information differed in all three scenarios. Results reported that trust and evaluation of information differed in all three scenarios. In the SARS-CoV-2 pandemic, there was a high increase in trust in the information provided ‘inside’ by the shopping location, while there were no changes for the ‘outside’; trust in cooperative stakeholders went from a medium-low to medium-high score, while, for individual stakeholders, it was maintained as a medium score, and trust in information on food products was kept at high score [8]. The authors indicate that, regarding the evaluation of the information provided by stakeholders, a tendency towards a maintaining a medium score was seen, while that from the channels of distribution went from medium-low to medium-high for buying on-site. A uniform tendency was observed for online/other distribution channels for all three years and descriptors studied: “Internet”, “Farmer on-demand”, and “Cooperative consumers” (<50%). In summation, Cantalapiedra et al. (2022) stated that their research provides findings of implications that contribute to the changing the perception of food risk, due to the COVID-19 pandemic, i.e., the adaptation of the young population, trust in safety and quality, and importance of coordination from all communication points to avoid negative or strong consequences, in case of future lockdowns or health crises [8].

Lastly, around food risk and safety perception in this collection, Li et al. (2021) investigated trends in food preferences and sustainable behaviour in Spanish consumers during the COVID-19 lockdown. The authors indicate that the research not only investigates trends in Spanish consumers’ general food shopping and consumption habits during the lockdown, but also investigates these trends from the perspective of sustainable purchasing [10]. Specifically, total food consumption (C), food expenditure (E), and purchase of food with sustainable attributes (S) were measured. Data were collected from a semi-structured questionnaire which was distributed online among 1203 participants. Li et al. (2021) describe how logit models showed that gender, age, employment status, and consumers’ experiences were associated with total food consumption and expenditure during the lockdown. In addition, Li et al. (2021) state that consumers’ risk perceptions, shopping places, trust level in information sources, and risk preference were highly essential factors influencing consumers’ preferences and sustainable behaviour. Consumers’ objective knowledge regarding COVID-19 was related to expenditure. Furthermore, family structure only affected expenditure, while income and place of residence influenced food consumption. Mood was associated with expenditure and the purchase of sustainable food. Household size affected purchasing behaviour towards food with sustainable attributes. Li et al. (2021) summaries that this research provides references for stakeholders that help them to adapt to the new COVID-19 situation [10].

### 2.4. Purchasing Behaviour and Decisions

The final grouping of works in the present collection covers purchasing behaviour and decisions as affected by the COVID-19 pandemic. Qi et al. (2021) looked at explaining Chinese consumers’ green food purchase intentions during the COVID-19 pandemic via an extended theory of planned behaviour [12]. The authors indicate that as a result of the COVID-19 pandemic, consumers’ habits and behaviours have been strongly influenced, potentially creating a more sustainable and healthier era of consumption. Hence, the authors conclude there is a potential for further expanding the green food sector in China [12]. Qi et al. (2021) present that the theory of planned behaviour (TPB) is a widely used framework to explain consumers’ food choices. The authors state that considering consumers’ internal norms, their perceptions of green food attributes, and the shifting consumer behaviour, their study has extended the TPB framework (E-TPB) by adding constructs of moral attitude, health consciousness, and the impact of COVID-19 (IOC). Qi et al. (2021) analysed the results of structural equation modelling among 360 functional samples, revealing that the E-TPB model has a superior explanatory and predictive power compared with the original TPB model regarding Chinese consumers’ green food buying intentions in the current and post-pandemic periods [12]. The authors’ path analysis (a form of multiple regression statistical analysis that is used to evaluate causal models by examining the relationships between a dependent variable and two or more independent variables) demonstrated that attitude, perceived behavioural control, moral attitude, health consciousness, and IOC have significant positive effects on green food purchase intentions. In conclusion, however, the authors state that the association between subjective norm and purchase intention varies within the TPB and E-TPB models, which showed a non-significant impact in E-TPB. Overall, Qi et al. (2021) indicate that these findings can generate more suitable managerial implications to promote green food consumption in China during the current and post-pandemic periods [12].

A second investigation in this space looked at COVID-19′s first wave, regarding an examination of the pandemic’s impact on food purchasing behaviour in the Eurozone [13]. Gutiérrez-Villar et al. (2021) present that COVID-19 has had a negative impact on the living conditions of people in all countries worldwide. With a devastating economic crisis where many families are finding it difficult to pay bills and make ends meet, increases in the prices of food basket staples can be very worrying. Their study examines the relationship between the incidence of the pandemic during the first wave in 16 Eurozone countries with the variation experienced in food prices [13]. Gutiérrez-Villar et al. (2021) analysed the harmonised index of consumer food prices (included in HICP) and the classification of the degree of pandemic impact by country, the latter established with the index of deaths provided by the Johns Hopkins Center [13]. The procedure the authors used compared actual food prices during the first wave (March to June 2020) with those foreseeable in the absence of the pandemic. Time series analysis was used, dividing the research period into two phases. Gutiérrez-Villar et al. (2021) indicated that in both phases, the Holt–Winters model was applied for estimation and subsequent prediction. After a contrast using Kendall’s tau correlation index, Gutiérrez-Villar et al. (2021) concluded that in the countries with the highest death rates during the first wave, there was a higher increase in food prices than in the least affected countries of the Eurozone [13].

Lastly, in this collection, Jun et al. (2022) looked at how customer decisions were influenced to use online food delivery services during the COVID-19 pandemic. The authors contend that despite the popularity of online food delivery systems in the food service industry, there have been few studies into customers’ decision-making process to use online food delivery services during the COVID-19 pandemic. Jun et al.’s (2022) study applied the technology acceptance model (TAM) to examine the factors affecting customers’ intention to use online food delivery services [9]. Authors results showed that perceived usefulness affects customer’s online food delivery usage directly and indirectly through customer attitude; enjoyment and trust are also key factors determining behaviour intention toward customer attitude using online food delivery services, and that there is a positive relationship between social influence and customer attitude in addition to a positive relationship between customer attitude and behaviour intention in the online food delivery service context [9]. Jun et al. (2022) conclude that these findings provide theoretical and managerial implications that contribute to the online food delivery service industry [9].

## 3. Conclusions

Overall, the research included in this Special Issue collection is diverse and covers a wide range of investigations in relation to the effects and implications of COVID-19 for the human senses, consumer preferences, appetite and eating behaviour. Studies are included from the fundamentals of appetite and the senses on to real world applicability regarding food choice, safety perception and purchasing behavioural change as a result of the COVID-19 pandemic and its various waves and lockdowns across the world.

The diverse nature of the studies included in this Special Issue emphasizes the importance and critical nature of the inclusion of the human senses and consumer preference and behaviour in relation to addressing the after effects of the COVID-19 pandemic and its implications.

An overall conclusion with respect to this article collection would be that the human senses, consumer acceptance, and preferences are core to future food design, with respect to understanding COVID-19’s effects on human perception effects on a global scale.
